# Development of a Dynamic Web Mapping Service for Vegetation Productivity Using Earth Observation and *in situ* Sensors in a Sensor Web Based Approach

**DOI:** 10.3390/s90402371

**Published:** 2009-03-31

**Authors:** Lammert Kooistra, Aldo Bergsma, Beatus Chuma, Sytze de Bruin

**Affiliations:** Wageningen University, Centre for Geo-Information, Droevendaalsesteeg 3, NL-6708 PB Wageningen, The Netherlands; E-Mails: aldo.bergsma@wur.nl; beatuschuma@yahoo.com; sytze.debruin@wur.nl

**Keywords:** Gross Primary Production, sensor networks, earth observation, Sensor Observation Service (SOS), web mapping service (WMS)

## Abstract

This paper describes the development of a sensor web based approach which combines earth observation and *in situ* sensor data to derive typical information offered by a dynamic web mapping service (WMS). A prototype has been developed which provides daily maps of vegetation productivity for the Netherlands with a spatial resolution of 250 m. Daily available MODIS surface reflectance products and meteorological parameters obtained through a Sensor Observation Service (SOS) were used as input for a vegetation productivity model. This paper presents the vegetation productivity model, the sensor data sources and the implementation of the automated processing facility. Finally, an evaluation is made of the opportunities and limitations of sensor web based approaches for the development of web services which combine both satellite and *in situ* sensor sources.

## 1. Introduction

To facilitate environmental resource management of intensively populated countries like the Netherlands, integrated information systems which are capable of real-time monitoring of fundamental processes in the environment, as well as providing vital hazard warnings, are required. Traditionally, sensor networks covering various geographical and temporal scales are an important source of information for this task. They allow vast amounts of relevant information to be collected with a high temporal frequency for a network of point locations that are remote, inaccessible, or lack the necessary resources to acquire such information in a different manner [1]. For example, ground water levels in the Netherlands are monitored through a network of 4,000 semi-automated groundwater wells [2]. Recent developments in the miniaturization of electronics and wireless communication technology will enhance the opportunities of sensor networks for real-time monitoring of the natural environment [3]. Next to *in situ* sensor networks satellite remote sensing systems are also a key source of information for many applications. Although space based sensors have a superb spatial coverage, they can frequently incur a significant data delivery latency, have a poor signal to noise ratio, and possess coarse resolutions. However, for a comprehensive monitoring system to provide timely information, a combination of *in situ* and space based sensors offers a synergetic configuration [4]. In an integrated approach, the sensor observations provide data and information; scientific models use these data and produce predictive results which are provided to end-users to assist the decision making process [5]. Although space-based and *in situ* sensor data have not been integrated in a fully self-consistent way, advanced technologies of today make it possible to pursue more integrated approaches to environmental resource assessment [4].

Although information technology is an important facilitator in this process, integrated information systems are often limited by interoperability problems due to individual components which cannot easily communicate with each other [6]. To overcome this problem, efforts at the sensor network level are required which deal with issues such as fusion of sensor data and interoperability among networks and their connections to information systems. This effort should not only pay attention to the technical facilitation but also should include organizational and standardization aspects. The concept of sensor webs as introduced by Delin in 2002 [7] “allows for the spatio-temporal understanding of the environment through coordinated efforts between multiple numbers and types of sensing platforms, including both orbital and terrestrial and both fixed and mobile.” Compared to sensor networks, sensor webs are unique in their feature that sensors communicate with each other, share information with other sensors and are aware of their environment. Communication between the sensor web and user can be in two directions: the user receives information from the sensor web but can also send instructions to it [8]. In an initiative called sensor web enablement (SWE), the Open Geospatial Consortium (OGC) has been developing a framework of open standards for exploiting web-connected sensors and sensor systems of all types [9]. The available services include access to sensor measurements, retrieval of sensor metadata, controlling sensors, alerting based on sensor measurements and automatic processing of sensor measurements. Although the developed SWE concepts are being applied in a broad range of environmental domains (e.g., hydrology [9,10], ecology [11,12], risk management [6,9,13]), only a limited number of studies [4] describe the combined use of space-based and *in situ* sensor sources in a sensor web based approach.

Monitoring of terrestrial plant productivity is one of the key parameters in environmental resource management as it provides information on potential food resources and sources of wood for construction, fabrication and fuel [14]. For example, early indicators of crop health status are very valuable because management decisions can be made both by farmers at the field level but also by governments at the regional level to mitigate the economic and social impacts of yield variability. In addition, as climate and terrestrial ecosystems interact with and influence each other, vegetation productivity is also used as indicator for climate change effects [15]. Plant productivity is calculated as Net Primary Productivity (NPP), the difference between Gross Primary Productivity (GPP) and plant autotrophic respiration (Ra), which is the net carbon fixed by vegetation through photosynthesis [16]. At the global scale, terrestrial plant productivity is one of the most-modeled ecological parameters, with models that differ markedly in approach and complexity often yielding comparable estimates [17]. For example, a global 8-day MODIS product (MOD17A2) is available which models GPP at a 1 km resolution using a light-use efficiency model [18]. However, for regional applications (e.g., monitoring crop productivity) both the spatial and temporal resolution of this product is too coarse. In addition, this product has been developed for a global scale which means that several of the input parameters of the estimation model do not account for the local heterogeneity of land use and meteorological parameters [19–21]. Increased availability of real-time sensor data at the local scale could increase the understanding and detection of vegetation status of heterogeneous landscapes. The added value of a sensor web based approach would be that multi-source sensor streams can be integrated in the model. Standardized modeling results can be presented to the end-user and will supply information on the spatial distribution of vegetation productivity both in the actual situation (nowcasting) and for the near future (forecasting) [22].

In this study we have developed a sensor web based approach which combines earth observation and *in situ* sensor data to derive regular products for vegetation productivity on a regional scale level. The approach is implemented in an automated processing facility which makes the products available through a dynamic web mapping service (WMS). Within the study a prototype application has been developed which provides daily maps of vegetation productivity for regional to national scale in the Netherlands. In the results section of this paper the spatial-temporal development of GPP over the Netherlands is presented. Finally, we assess the validity of the modeling results and discuss the limitations and opportunities for further development of the presented methodology.

## 2. Materials and Methods

### 2.1. Modeling of vegetation productivity

During the last 20 years, several remote sensing based approaches have been developed to estimate vegetation productivity from global to regional scales [16]. The main concept for these approaches refer to experiments of Monteith [23] which showed that increase of plant biomass from well drained crops can be represented by the following equation:

(1) GPP=FPAR×LUE

where GPP is the gross primary production (gC m^−2^ day^−1^), FPAR is the fraction of absorbed photosynthetically active radition (unitless) and LUE is an empirical light use efficiency factor (gC MJ^−1^). Sellers [24] and Asrar [25] have shown that FPAR can be derived from remote sensing data by using the normalized difference vegetation index (NDVI) which uses the reflectance (ρ) for the red (RED) and near-infrared (NIR) wavelength:

(2a) $$\mathrm{NDVI}=\frac{\rho_{\mathrm{NIR}}-\rho_{\mathrm{RED}}}{\rho_{\mathrm{NIR}}+\rho_{\mathrm{RED}}}$$

and

(2b) $$\frac{\mathrm{FPAR}}{\mathrm{PAR}}\alpha \;\mathrm{NDVI}$$

where PAR is the incoming photosynthetically active radiation (MJ·m^−2^·day^−1^). Thus, spectral vegetation indices like the NDVI have a strong link to the fraction of photosynthetically active radiation that is absorbed (FPAR). When the FPAR is derived from NDVI and driven by the photosynthetically active radiation over a certain time step and converted by the LUE, the biomass production over this time step can be expressed as [26]:

(3) $$\text{GPP}=\text{NDVI}\times \text{PAR}\times (\text{LUE}\times S_{\text{Tmin}}\times S_{\text{VPD}})$$

where the scalars (unitless) for minimum temperature (S_Tmin_) and water availability (S_VPD_) are used to reduce the potential LUE to the actual efficiency [19]. LUE depends on the weather conditions which control the opening and closing of leaf stomata. Therefore, temperature and water stress were taken into account in the model.

**Figure 1.**
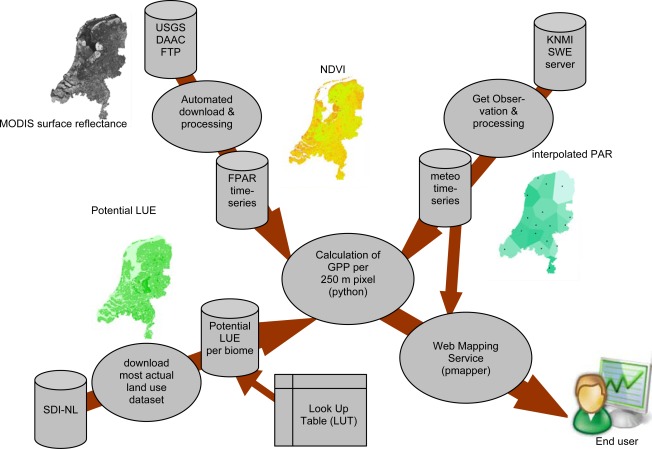
Overview of different steps in the automated processing chain for the calculation of vegetation productivity over the Netherlands and the presentation of resulting maps for GPP in web mapping service.

Both earth observation and *in situ* sensor data sources were used for the parameterization of the variables of the regional scale GPP model (equation 3). Figure 1 gives an overview of the paths for the input datasets and the intermediate products for calculation of daily GPP for the surface area of the Netherlands. The NDVI as proxy for FPAR (equation 2b) was derived from daily remote sensing data provided by the medium-resolution sensor MODIS on the Terra platform. The MODIS surface reflectance product (MOD09) was used as input for calculation of NDVI. The required meteorological data for 16 stations in the Netherlands were taken from the KNMI SWE server hosted by the Royal Dutch Meteorological Institute (KNMI). Several studies have shown that maximum LUE (equation 3) varies for different vegetation types [27]. Therefore a look-up-table (LUT) was used to set biome-specific parameters related to the estimation of the actual LUE. By combining the biome-specific LUT with a remote sensing based land cover database, the spatial distribution of LUE related parameters could be established. Finally, the whole procedure was implemented as an automated processing chain which calculates a daily 250 m GPP product and makes the resulting maps available to the end-users through a dynamic web mapping service. In the following paragraphs, the different parts of the processing chain as presented in Figure 1 will be explained in more detail.

### 2.2. Remotely sensed data

MODIS 250 m daily surface reflectance data acquired by the TERRA/AQUA platform (Product MOD09GQ) were downloaded from the USGS Land Processes DAAC ftp download facility (Figure 1). The MODIS Reprojection Tool was used to reproject the downloaded MODIS tile to UTM zone N31 and a spatial subset to the extent of the borders of the Netherlands was made. From the original HDF file format as provided by USGS, band 2 (reflectance in red: 620–670 nm), band 3 (reflectance in NIR: 841–876 nm) and band 4 (band quality) were stored in geotiff file format. To facilitate further processing, the geotiff file format was converted to ASCII raster using the GDAL translator library (http://www.gdal.org). To mask out the pixels which were completely covered or partly affected by clouds, information from the reflectance band quality was used. Only pixels which were flagged as being correctly produced with ideal quality for all bands (code 00) were further processes. Pixels with other quality codes were set to a no data value. During processing of the images, we found that not all clouded pixels were removed. Therefore, an additional masking step was applied: all pixels with a value smaller than 0 or larger than 1,500 for MODIS band 2 were set to a no data value. For the remaining pixels in the image, NDVI was calculated according to equation 2a and used as estimate for FPAR.

### 2.3. Meteorological data

To investigate the feasibility of sensor web enablement (SWE) for operational management of meteorological sensors within the Royal Dutch Meteorological Institute (KNMI), a KNMI SWE server was established. Through this SWE server data for nine meteorological parameters of 16 stations in the Netherlands was made available starting from August 2007 onwards. Real time data from the KNMI Meteorological Data distribution System (KMDS) are exported through a ftp server and a feeder to an independent PostGIS database. Because the KMDS database is used in an operational environment all SWE related operations were carried on the PostGIS database which is hosted at the KNMI SWE server. This database stores both the observation values and corresponding meta data. Using a Sensor Observation Service (SOS) [28], available data and metadata can be accessed, queried and downloaded by using the operations DescribeSensor, GetCapabilities and GetObservation, respectively. The SOS is one of a family of standards and specifications that make up the OGC Sensor Web Enablement activity. Queries to and replies from the SOS are XML based and require a XML viewer for further processing. The implementation of the KNMI SWE server was based on the open source software provided by the 52°North Initiative (http://52north.org).

The required meteorological parameters for the vegetation productivity model (equation 3) were acquired from the KNMI SWE server using the SOS GetObservation request. No direct measurements for PAR which designates the spectral range of solar light between 400 to 700 nm were available from the SWE server. Instead PAR was derived from the measured parameter global solar radiation which is the total incoming radiation between 300 and 3,000 nm. In the literature [29] several constants dependent on latitude and cloud conditions are reported to transform global solar radiation to PAR. The reported values vary around 0.45 which was used for the transformation of global radiation to PAR in this study. The 10 minute instantaneous measurements over the day were transformed to an average daily PAR value and used as input for the vegetation productivity model.

For the calculation of the two scalar values of the vegetation productivity model, S_Tmin_ and S_VPD_, additional time-series for air temperature (at 1.5 m in °C) and relative humidity (at 1.5 m in %) were requested from the KNMI SWE server. The minimum temperature over a day was derived from the 10 minute air temperature time-series and used as input for calculation of the S_Tmin_ scalar. Both the air temperature and relative humidity were adopted to calculate the S_VPD_ according to method described by Choudhury [30]. Continuous 250 m ASCII rasters for the three meteorological parameters PAR, S_Tmin_ and S_VPD_ were prepared by using Thiessen polygons as spatial interpolation method.

### 2.4. Biome-specific Light Use Efficiency

Light Use Efficiency is a key parameter for the estimation of vegetation productivity, but varies widely for different biomes or vegetation types [27]. Two main sources of this variation can be distinguished. First, biomes differ in their vegetation physiology, which results in varying efficiencies in photosynthesis and respiration. In addition, individual studies have suggested that also within biomes variation is present due to factors such as stand age, species composition, soil fertility and foliar nutrients [31]. In this study, we accounted for differences between biomes by assigning biome specific LUE values (Table 1) derived from the work of Gower *et al*. [31]. Secondly, variability of LUE is due to suboptimal climate conditions. In general, LUE is attenuated by two main controls: stomatal closure due to cold night temperature (Tmin) and stomatal control due to daytime vapor pressure deficiency (VPD) [18]. Both controls were included in the vegetation productivity model (equation 3) using a linear scalar approach [32]. This means for example for Tmin when the temperature is below a certain threshold (Tmin_min_; Table 1) the scalar is set to 0 and the resulting actual LUE is 0. Above a certain upper minimum temperature threshold (Tmin_max_; Table 1) no reduction of LUE is accounted for. For the temperature range between the two thresholds a linear transformation was applied. The same methodology was also applied for VPD. Real-time meteorological data from the KNMI SOS were used for calculation of daily scalar values for Tmin and VPD and minimum and maximum thresholds (Table 1) were derived from the work of Heinsch *et al*. [32].

The Dutch Land Cover Database (LGN4) [33] was used to derive the spatial distribution of the relevant biomes (Table 1) over the Netherlands (Figure 1). The original 39 land use classes were translated to five main biome classes for the Netherlands as presented in Table 1. Next to the vegetation biome classes, three classes without vegetation productivity were distinguished: water, urban and built-up area and bare land. For these classes no productivity estimations were made, but they were used for visualization purposes of the final mapping product. The original LGN pixel resolution of 25 m was aggregated using majority rule to 250 m in order to match the pixel resolution of the MODIS images. The resulting biome map was linked with the biome specific potential LUE values presented in Table 1 which were assumed static over the year. Calculation of actual LUE according to equation 3 was achieved by combining the potential LUE map with interpolated maps for Tmin and VPD and applying the scalar thresholds as presented in Table 1.

### 2.5. Implementation of automated processing facility

To make the daily processed vegetation products easily available to a broad range of potential end-users, a dynamic web mapping service (WMS) was developed. In addition, to provide ‘near’ real time products, the different steps of the procedure were implemented as automated processing chain. The whole procedure consists of different modules (Figure 1) and the object-oriented programming language python (http://www.python.org) was adopted to link the activities for the different modules. The following processing steps are carried out in the automated processing chain:

- The MODIS surface reflectance product (MOD09QC) is downloaded from the USGS Land Process DAAC data pool on a daily basis. The data pool provides direct ftp access to the most recent MODIS products (ftp://e4ftl01u.ecs.nasa.gov/MOLA/MYD09GQ.005). The MODIS Reprojection tool is used to clip and reproject the images and the GDAL tool is used to convert the images from GeoTiff format to ASCII raster;
- Masking cloud contaminated pixels by checking MODIS band quality (band 4) or thresholds of red band (band 2) and calculation of NDVI (equation 2a) as proxy for FPAR as 250 m ASCII raster;
- Meteorological data are requested from the KNMI SWE server on a daily basis using the SOS GetObservation operation. After processing of the data, observations for 16 stations are interpolated using Thiessen polygons, resulting in 250 m ASCII rasters for PAR, S_Tmin_ and S_VPD_;
- Potential LUE derived from the aggregated biome map is stored as static grid file (ASCII raster) with 250 m resolution;
- Combining the different intermediate products, a per pixel calculation is made for all vegetation covering pixels according to equation 3, resulting in the final GPP product;
- The final mapping products are stored as ASCII raster and made available through a WMS. We used the Open Source platform UMN Mapserver (http://mapserver.gis.umn.edu/) together with p.mapper (http://www.pmapper.net) for implementation of the WMS. The Mapserver platform serves as common gate interface which supports a whole range of OGC and ISO standards. The p.mapper framework provides a suite of standardized functionality for viewing, query and processing of spatial data.

The automated processing chain has been implemented for the surface area of the Netherlands. In this paper results for vegetation productivity for the year 2008 between January 1 and December 31 are presented. The dynamic web mapping service will be continued after this date.

## 3. Results

### 3.1. Dynamic web mapping service

**Figure 2.**
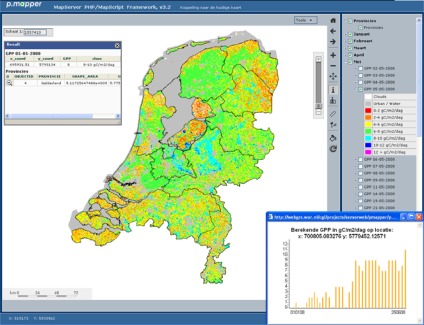
User interface of the dynamic WMS for spatio-temporal development of vegetation productivity over the Netherlands. Vegetation productivity is expressed as GPP in gC m^−2^ day^−1^.

The developed dynamic WMS (WMS access at: http://webgrs.wur.nl/cgi/projects/sensorweb/pmapper/pmapper_gpp/map.phtml.) for vegetation productivity (Figure 2) enables end-users to visualize and analyze the spatio-temporal development of GPP over the Netherlands. Standard functionality of the WMS (e.g., zooming and panning, measure distance, make layers transparent, printing and downloading) is provided through the p.mapper framework. Individual vegetation productivity layers for a specific date can be downloaded in GeoTiff format and combined with other relevant spatial data. In addition, some dedicated functionality has been added to the WMS in order to visualize actual changes in vegetation productivity:

- Information on most recent vegetation productivity: after selection of a pixel, actual values of vegetation productivity are listed for all opened layers of the WMS (Figure 6: upper left);
- Trajectories of vegetation productivity: after selection of a pixel, the time-series of vegetation productivity of all available dates for this pixel is presented for the most recent year available (Figure 6: lower right).

### 3.2. Development of GPP over the Netherlands in 2008

**Figure 3.**
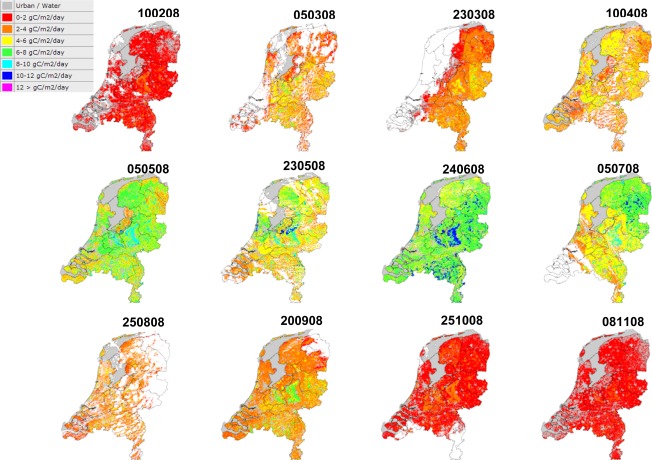
Monthly development of GPP (gC m^−2^ day^−1^) over the Netherlands for the year 2008. The numbers above the maps refer to the date (ddmmyy) for which the map was produced.

Based on the stored vegetation products in the dynamic WMS, an analysis was made of the spatio-temporal development of GPP over the Netherlands in 2008 (Figure 3). The analysis clearly shows the phenology of the vegetation with low productivity at the end of the winter in February (GPP < 2 gC·m^−2^·day^−1^) and increasing productivity at the start of the growing season in March and April. Differences between biomes over the season are observed with especially relatively high productivity values for the large-scale nature reserve the Veluwe in the centre of the Netherlands (e.g., dates 240608 and 200908 in Figure 3). In addition, the late arable crops in the Flevopolder along the IJsselmeer can clearly be observed (red/orange colors due to bare soil) from the vegetation productivity map of the 5^th^ of May (Figure 3). Maximum productivity occurs at the end of June after which the productivity is reducing again till winter levels at the end of October.

Within Figure 3 several disturbance can be observed on the spatial distribution of vegetation productivity. First of all, cloud coverage reduces for some dates the productivity estimation over the complete surface area of the Netherlands. This can either be a large continuous area (e.g., date 230308 in Figure 3) or smaller scattered areas (e.g., date 250808 in Figure 3) depending on the cloud coverage at the time of acquisition of the MODIS image. In addition, the effect of a relatively low number of meteorological stations on the quality of spatial interpolation can be observed in the productivity map for March 23. This results in some sharp linear boundaries between vegetation productivity classes which in reality probably will not be present.

**Figure 4.**
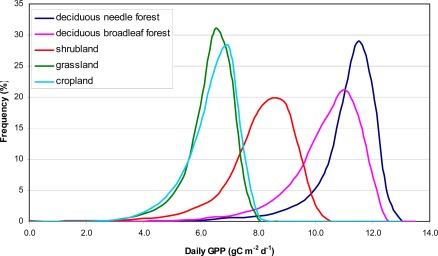
Frequency distribution for daily GPP (gC·m^−2^·day^−1^) per biome type over the Netherlands for June 24, 2008.

**Figure 5.**
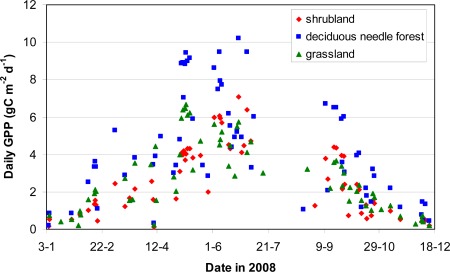
Time-series comparison for daily values of GPP (gC·m^−2^·day^−1^) in 2008 for selected locations in three different biomes.

Within a vegetation map for a specific day, there is a considerable difference in the spatial distribution of GPP for different biomes. Figure 4 shows the distribution of all calculated GPP values per biome over the Netherlands for June 24 which is around maximum productivity. The graph shows a clear differentiation between biomes which for a large part is driven by the defined potential LUE per biome (Table 1). The variability around the maximum GPP value per biome (Figure 4) is caused by spatial variation in meteorological parameters and FPAR. The temporal development of GPP over the year also shows clear differences between biome types (Figure 5). Deciduous needle forest has a relatively higher productivity compared to shrubland and grassland. The influence of FPAR on the estimated GPP can clearly be observed by the difference for the biomes grassland and shrubland (Figure 5) in start of growing season (respectively, March and April) and the timing of maximum productivity (respectively, May and July). The relatively late development of vegetation productivity for shrubland is caused by the delayed phenology of the heath land vegetation which is the main vegetation type present in the shrubland biome.

Figure 5 shows that although there is a clear trend in development of GPP, also a considerable scatter around this trend is present. This scatter is mainly caused by day-to-day variations in weather resulting in considerable variation in PAR. During the growing season the influence of S_Tmin_ will be limited while the S_VPD_ will influence the productivity during dryer periods. Due to a long period of clouded conditions at time of overflight of the MODIS sensor in the month of August only a limited number of observations was available for this month (Figure 5). On average per pixel between 65 and 75 days over the year were non-clouded and thus for those days daily GPP could be estimated using optical remote sensing based measurements.

## 4. Discussion

### 4.1. Evaluation and validation of GPP model

**Figure 6.**
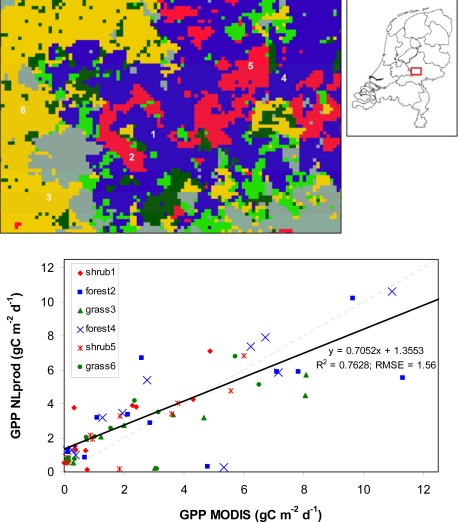
Comparison of 8-day MODIS GPP product (MOD17A2) with daily GPP estimation from this study for three biome types at six locations located in the Veluwe nature reserve in the centre of the Netherlands (see inset). A comparison was made for the 12 monthly dates as presented in Figure 3.

From an end-user perspective, a statement on the quality of the product provided through a WMS is as important as the product itself. Therefore, documentation of product quality is an essential step to improve the usability of integrated products derived using sensor based data sources. Estimates of GPP are in general validated using eddy flux tower data which provides flux measurements on carbon and water exchange from the land surface to the atmosphere within the footprint of the tower [19]. Within the scope of this study these data were not available yet. As an alternative we have compared daily GPP as derived from the automated processing facility with GPP estimates from the 8-day MODIS GPP product (MOD17A2) as provided through the USGS Land Process DAAC data pool (Figure 6).

For the comparison, 12 MODIS GPP products corresponding to the monthly dates of the GPP products as presented in Figure 3 were downloaded. For the biome types deciduous needle forest, shrubland and grassland, we selected two locations for each biome in the central part of the Netherlands with a relatively large homogenous surface area (Figure 6). For the six selected locations, the estimated GPP derived from the MODIS product was compared to the estimated GPP as derived within the present study (Figure 6). In general the GPP values show a good agreement with an R^2^ of 0.76 over all biome types, excluding estimates from the data in April for which relatively low values were obtained for the daily-based GPP from this study. The latter can be related to relatively cloudy conditions at time of overflight of the MODIS sensor at that day. Because the MODIS GPP product is derived from data over eight days, probably data from another day in this period will have been used for that calculation. Estimates of GPP for grassland from this study show a small underestimation compared to the MODIS GPP product, while daily GPP values for shrubland are overestimated (Figure 6). This difference can mainly be attributed to the relatively large uncertainty in the determination of LUE [19]. Earlier studies have indicated the use of alternative approaches to derive LUE for example using the photochemical reflectance index (PRI) [5], however this methodology is not yet operational from spaceborne platforms.

Annual GPP estimates derived from this study and the MODIS GPP product were compared to annual GPP figures for the Netherlands available from literature (Table 2). Only a limited number of studies on GPP development for the Netherlands were available [34,35]. Based on this first limited assessment (Table 2), it was observed that annual GPP values for deciduous needle forest from this study agreed well with measured annual GPP (Table 2). Annual GPP estimates for grassland from both this study and from the MODIS product fall outside the measured range of eight Dutch grassland sites [35]. As already indicated uncertainty in determination of LUE plays an important role in this and further study is therefore required to improve the model results for this biome. Complete modeling of the carbon balance of the biomes can be achieved by taking into account the processes of autotrophic and heterotrophic respiration and carbon removal due to harvest or other disturbances [19]. However, for this study we used a relatively simple productivity model, as the focus was on the development of a complete chain approach from individual sensors to the final end user.

### 4.2. Limitations and opportunities for sensor web based approach

Within this study we have developed an automated processing facility to derive products relevant for environmental resource management from multi-source sensor data using a sensor web based approach. Interoperability between sensor data streams and connection with the information system was achieved by using open-source implementations of international standards for SWE and WMS. Use of common standards is an important requirement for upscaling of the developed facility, both in terms of number of sensors and inclusion of new sensor types. The interoperability of the presented processing facility could be further improved by using Web Processing Services (WPS) and Web Coverage Services (WCS) for interpolation of point data and access to gridded surfaces (e.g., satellite images) respectively [36]. Furthermore, additional functionality (e.g., temporal zonal statistics) in the WMS would be helpful to query and visualize the dynamic dimension of the products derived from the automated processing chain. Unfortunately, only a limited number of sensor web based meteorological stations was available. This is mainly related to the fact that the OGC SWE standards have only recently become available and most organizations responsible for *in situ* sensor networks haven’t yet introduced this in their actual processing facility. The expectation is that in the coming five to 10 years this will change and a large number of sensor nodes in the currently running geo-sensor networks (meteorology, groundwater etc.) will be operated within a standardized SWE based concept. In addition, dedicated networks (e.g., protection of dikes) which are newly developed will more quickly be adapted to newly available technology. However, in order to discover available sensors and sensor data, which take into account the dynamic behavior of the sensors, new approaches for sensor discovery need to be developed [37].

At this moment, actual processing and visualization of vegetation productivity within the developed facility is not real time yet but has a delay up to 10 days. Main reason is the time delay due processing and archiving of the MODIS images from the USGS Land Processes DAAC ftp download facility. This could be reduced by downloading raw unprocessed MODIS data (available within one day). However this would require development of a hardware and software set-up for complete (automated) processing of satellite data from raw data to surface reflectance. Another important limitation in the use of optical remote sensing data for daily monitoring is the problem of cloud coverage. For the Dutch situation, on average between 40 and 60 days per year have a cloud coverage smaller than 30% for the Netherlands during the MODIS overpass. As a result it will not be possible to produce daily maps of vegetation productivity with a complete coverage and also the frequency of coverage per pixel will be variable. For the Dutch situation with its relatively heterogeneous landscapes and short-scale variability, the medium resolution of 250 m to 500 m for MODIS data will often be to coarse. However, higher resolution satellite based remote sensing sources (10–30 m) are only limited available (Landsat, SPOT). In that respect, recent developments in the field of remote sensing data fusion [38,39] could be of interest to improve the spatial resolution to relevant management units (< 30 m).

Recent developments within NASA and ESA are aiming at provision of real-time earth observation products to the end-users, so the expectation is that within the coming years, real-time availability of earth observation data and products will improve. Within ESA, several projects [40] are dealing with the use of (OGC) SWE technology to connect *in situ* sensor webs with remote sensing sensors which is brought together in the ESA Service Support Environment. Within NASA, fast delivery of EO-1 remote sensing products has initiated several projects which use SWE to control and access earth observation sensors to monitor the development of hurricanes and wildfires [41].

## 4. Conclusions and Outlook

In this article we have presented a sensor web based approach which combines earth observation and *in situ* sensor data to derive near real-time vegetation productivity products. A prototype application for monitoring GPP over the Netherlands was successfully developed and implemented within an automated processing facility. Continuous GPP maps are provided to the user through a web mapping service which not only provides functionality for spatial analysis but also includes functionality to present time-series for selected locations.

In order to achieve an added value for end-user applications using increasingly available real time earth observation data, they need to be combined with *in situ* sensor data and environmental models to derive higher level products relevant for environmental resource management. In this study, interoperability between sensor data streams and connection with the information system was achieved by using open-source standards for SWE and WMS. For example, meteorological data were obtained in a standardized way through a Sensor Observation Service as developed within OGC-SWE. Use of common standards is an important requirement for upscaling of the developed facility, both in terms of number of sensors and inclusion of new sensor types.

Further development of the presented approach will focus on the establishment of an integration platform for near real-time assimilation of sensor data sources into simulation models. Focus will be on integration of multi-source sensor data streams and the opportunities for remote sensing data fusion to improve spatial resolutions to relevant management units (< 30 m).

## Figures and Tables

**Table 1.** Biome Look Up Table for calculation of potential light use efficiency to actual value.

**Biome**	**Potential LUE (gC MJ^−1^)**	**Tmin_min_^1^ (°C)**	**Tmin_max_^1^ (°C)**	**VPD_min_^2^ (Pa)**	**VPD_max_^2^ (Pa)**
**Deciduous needle forest**	1.103	−8.00	10.44	650	3100
**Deciduous broadleaf forest**	1.044	−8.00	7.94	650	2500
**Shrubland**	0.888	−8.00	8.61	650	3100
**Grassland**	0.680	−8.00	12.02	650	3500
**Cropland**	0.680	−8.00	12.02	650	4100

^1^ refers to minimum and maximum threshold for scalar S_Tmin_ (equation 3);

^2^ refers to minimum and maximum threshold for scalar S_VPD_ (equation 3).

**Table 2.** Comparison of annual GPP (gC·m^−2^·day^−1^) estimates for different biomes in the Netherlands.

**Biome**	**Annual GPP^1^: this study**	**Annual GPP^1^: MODIS product**	**Annual GPP: literature**	**Source**
**Deciduous needle forest**	1564 – 1816	1692 – 1838	1559	Dolman et al. [34]
**Grassland**	990 – 1057	885 – 1152	1300–1350^2^	Jacobs et al. [35]

^1^ refers to values for two selected locations (Figure 6);

^2^ refers to value for organic and mineral soils, respectively.
